# Impulsivity, Gambling-Related Cognitions, Cognitive Reappraisal and Gambling Behaviour in a Malaysian Sample

**DOI:** 10.1007/s10899-023-10246-7

**Published:** 2023-08-07

**Authors:** Gillian Shu Lin Tan, Cai Lian Tam

**Affiliations:** Department of Psychology, Jeffrey Cheah School of Medicine and Health Sciences, Monash University Malaysia, Jalan Lagoon Selatan, Bandar Sunway, Subang Jaya, Selangor 47500 Malaysia

**Keywords:** Gambling, Impulsivity, Cognitive distortions, Cognitive reappraisal, Moderation

## Abstract

The relationships between cognitive reappraisal and problem gambling have been widely studied in different contexts. However, previous research findings remain inconsistent. This discrepancy might be attributed to the effects of interactions between cognitive reappraisal and other risk factors for problem gambling. Using moderation models, this study examined the association between impulsivity, gambling-related cognitive distortions, cognitive reappraisal and problem gambling in a sample of Malaysian gamblers. A total of 149 community gamblers (103 males, 46 females; mean age = 32.18) completed an online questionnaire. Problem gambling was measured with the South Oaks Gambling Screen (SOGS); cognitive reappraisal was measured using the Emotion Regulation Questionnaire-Cognitive Reappraisal Subscale (ERQ-CR); impulsivity was measured with the Short-UPPS-P Impulsive Behaviour Scale (SUPPS-P); and gambling-related cognitive distortions were measured using the Gambling Related Cognitions Scale (GRCS). The results revealed impulsivity and gambling-related cognitive distortions as significant predictors of problem gambling. At high levels, impulsivity and cognitive distortions are significant moderator variables that strengthen the association between cognitive reappraisal and problem gambling. These findings demonstrate that reappraisal skills could exacerbate problem gambling severity amongst impulsive or self-deceptive gamblers. Future research with larger and more representative samples is needed to validate and generalise these findings.

## Introduction

Disordered gambling is a recognised mental health disorder characterised by difficulties controlling gambling spending, chasing losses, dishonesty about gambling, and severe negative outcomes associated with excessive gambling (American Psychiatric Association, 2013). Problem gambling, on the other hand, is a broader term that encompasses subclinical conditions where individuals face significant adverse consequences due to gambling (Gainsbury et al., 2014). This term is commonly used in studies that employ screening measures to identify problem gamblers without confirming their condition through clinical interviews and diagnosis. Nevertheless, problem gambling research often includes disordered gamblers as well.

The prevalence of problem gambling in Malaysia has seen a rise in recent years (Rathakrishnan & George, 2020). Utilising a proportionate stratified random sampling method and Problem Gambling Severity Index (Ferris & Wynne, 2001) as a community prevalence estimate, Loo and Ang (2013) found that 4.4% and 10.2% of the general Malaysian population engaged in high-severity problem gambling and moderate-severity problem gambling. These findings put Malaysians on the higher end of the gambling spectrum when compared to other Asian populations (Fong & Ozorio, 2005; Wong & So, 2003). A rising trend in problem gambling is concerning as it has great costs at both the individual and societal levels as it could lead to conflicts in relationships, work, finances and even potential legal issues. It is common for intimate partners of problem-gambling individuals to grapple with severe mental, emotional and financial stress (Rathakrishnan & George, 2020). The intergenerational transmission of problem gambling also puts children with problem gambling parent(s) at a much higher risk of developing problem gambling themselves (Dowling et al., 2018).

### Common Risk Factors for Problem Gambling

Previous studies have identified impulsivity as a risk factor for problem gambling with robust findings. Facets of impulsivity that have been repeatedly demonstrated in empirical studies to distinguish problem gambling individuals from healthy controls best are high levels of positive urgency (i.e. the tendency to act rashly when in an unusually positive mood), high levels of negative urgency (i.e. the tendency to act rashly in response to distress), and low levels of perseverance (i.e. the tendency to quit when a task becomes boring or difficult; Leppink et al., 2016). Longitudinal studies have also ascertained the temporal relationship between developmental trajectories of impulsivity and problem gambling. Liu et al. (2013) found that young adolescents with high impulsivity trajectories have three times the odds of developing problem gambling compared to their low impulsivity counterparts during emerging adulthood. Likewise, Dussault et al. (2011) explored the longitudinal relationships between problem gambling, impulsivity and socio-family risks. Over the course of ten years, impulsivity is the only significant predictor of problem gambling.

Another common risk factor for problem gambling is gambling-related cognitive distortions. Gambling-related cognitive distortions are defined as dysfunctional and erroneous beliefs surrounding gambling abilities and outcomes (Raylu & Oei, 2004b). It is classified into five categories: gambling expectancies (i.e. the perceived expectations about the effects of gambling), illusion of control (i.e. the perceived ability to control gambling outcomes), predictive control (i.e. the perceived ability to predict gambling outcomes), inability to stop gambling (i.e. the belief that one has difficulty or is unable to stop engaging in gambling behaviours), and interpretive bias (i.e. the cognitive process in interpreting and reframing gambling outcomes). Empirical studies have found a positive association between gambling-related cognitive distortions and problem gambling (Mitrovic & Brown, 2009; Orgaz et al., 2013). Meta-analysis also concluded that gambling-related cognitive distortions are an important component of problem gambling with a large effect size (Goodie & Fortune, 2013).

### Cognitive Reappraisal in Problem Gambling

In gambling research, cognitive reappraisal is a form of emotion regulation skill that plays a pertinent yet divisive role. Traditionally regarded as an adaptive strategy (Navas et al., 2016), cognitive reappraisal allows an individual to reinterpret an emotion-eliciting event via the modification of its attached meaning and the modulation of its emotional impact (Cutuli, 2014). In the emotion-generative process (Gross, 2002), cognitive reappraisal is conceptualised as an antecedent-focused strategy that should occur prior to the activation of an emotional event. Cognitive reappraisal would be applied to reappraise potentially distressing stimuli prior to any emotional or behavioural response. If effectively applied, cognitive reappraisal has the potential to alter the temporal course of the emotion-generative process by pre-empting a full-blown emotional response (Gross, 2002). Moreover, as cognitive reappraisal takes place in the initial stages of the emotion-generative process, its application is often spontaneous and occurs implicitly (Kobylińska & Kusev, 2019; Lambie & Marcel, 2002).

Numerous studies have found significant associations between cognitive reappraisal and higher satisfaction in interpersonal relationships (Srivastava et al., 2009), fewer symptoms of psychopathology (Aldao et al., 2010), and enhanced well-being (Aldao et al., 2010). Yet, this apparent congruity in literature does not extend to gambling research. To illustrate, Yang et al. (2013) studied the application of cognitive reappraisal strategy in response to gains and losses. The researchers found that cognitive reappraisal has an effect on an individual’s motivation to gamble. Specifically, it reduces the emotional experiences associated with losing money during gambling activities. Nevertheless, this was an experimental study conducted with non-gambling participants who were given a monetary gambling task. Hence, the findings might not extend to the problem gambling population. Alternatively, Navas et al. (2016) utilised a case-control study design and found significant associations between gambling disorder and emotion regulation strategies. Their findings suggest that compared to healthy controls, gambling disorder patients apply emotion regulation strategies that are regarded as adaptive to down-regulate the negative emotions and decrease the impact of losses due to gambling. Nevertheless, this study was conducted on individuals with a gambling disorder diagnosis, and their findings might not apply to problem gamblers with lower gambling severity.

Although the two aforementioned studies (Navas et al., 2016; Yang et al., 2013) provided evidence for the association between cognitive reappraisal and problem gambling. Discrepancies were noted in other studies (Pace et al., 2015; Williams et al., 2012) that obtained differential results. These studies attributed gambling behaviour to deficits in emotion regulation skills and argued that gamblers gambled to regulate their emotions as it provides a temporary escape from negative emotions—negative emotions that could have been effectively addressed if cognitive reappraisal strategies were utilised. For example, comparing 88 low-risk gamblers to 100 problem gamblers, Pace et al. (2015) found that problem gamblers had significant deficits in cognitive reappraisal skills. Further probing of the relationship between gambling behaviour and cognitive reappraisal also revealed that the negative association was significant. Results from Williams et al.’s (2012) study comparing 56 problem gamblers to matched controls also indicated that problem gamblers have significantly lower cognitive reappraisal scores. However, unlike Pace et al. (2015), Williams et al. (2012) did not find a significant association between problem gambling and cognitive reappraisal. Similarly, two recent meta-analyses (Neophytou et al., 2023; Velotti et al., 2021) also failed to identify a significant effect in the relationship between problem gambling and cognitive reappraisal.

In short, research on the direction and significance of the relationship between cognitive reappraisal and problem gambling is fraught with inconsistencies. Some papers (Navas et al., 2016; Ruiz de Lara et al., 2019) demonstrated that cognitive reappraisal has a positive association with problem gambling as it allows gamblers to cope with negative gambling outcomes. Conversely, other studies (Pace et al., 2015; Williams et al., 2012) found negative associations between cognitive reappraisal and problem gambling. Moreover, further discrepancies were also noted regarding the significance of these relationships (Neophytou et al., 2023; Velotti et al., 2021).

Although the role of cognitive reappraisal in problem gambling evokes much discourse, Velotti et al. (2021) cautioned against the examination of associations between constructs of emotion regulation and problem gambling *vis-à-vis* the *a priori* assumptions that emotion regulation strategies are adaptive or maladaptive per se. Instead, the adaptive potential of emotion regulation strategies might be better assessed whilst considering contextual factors and the effects of moderators.

### Moderators in Problem Gambling: Impulsivity

With reference to the coping theory (Lazarus & Folkman, 1984), addictive behaviours are often associated with negative emotion-focused coping, such as avoidance or escapism, i.e., the facet of negative urgency in trait impulsivity. In contrast, positive emotion-focused coping, such as positive reappraisal and mindfulness, entails the effective regulation of one’s emotions and, thus, is strongly associated with having high emotion regulation skills (Calado et al., 2017). Neuroscience research also provides further evidence for the association between impulsive behaviours and emotion regulation. Brain imaging studies have uncovered that the prefrontal cortex, particularly the medial prefrontal cortex and the amygdala, are responsible for controlling impulses (Kim & Lee, 2010) and regulating emotions (Ray & Zald, 2012). Dysfunction in this brain region could also cause severance of the cognitive control functions from the limbic system, resulting in emotional lability and impaired impulse inhibition (Churchwell & Kesner, 2011). As both impulsivity and emotion regulation are related to the coping theory and overlap in their neural circuitry, it would be interesting to study how impulsivity might moderate the association between cognitive reappraisal and problem gambling.

Research on the difficulties in managing emotions and the use of maladaptive coping strategies has found the association to be exacerbated by poor impulse control (Baumeister et al., 2000). Individuals with high impulsivity struggle more to maintain self-control, which could deplete the resources required for effective emotion regulation, putting them at an increased risk of disinhibited behaviour (Baumeister et al., 2000). Having high impulsivity also predisposes an individual to act on their impulses before giving themselves a chance to reappraise the situation instead, which could aggravate their addiction (Williams et al., 2012). To our knowledge, no studies have examined the moderating effect of impulsivity on cognitive reappraisal and problem gambling. As such, findings from this paper will attempt to address this gap in literature.

### Moderators in Problem Gambling: Gambling-Related Cognitions

The Gambling Space Model (GSM; Navas et al., 2019) proposed that putatively adaptive forms of emotion regulation, such as cognitive reappraisal, could be utilised by problem gamblers to defend their excessive gambling behaviours. The GSM postulates that through a process of cognitive elaboration, reappraisal skills can manifest as self-deceptive reasoning, which aids problem gamblers in coping with accumulated gambling losses via the distortion of reality in a self-serving way (Ruiz de Lara et al., 2019). Furthermore, positive reappraisal might also be applied to negative gambling events as motivated reasoning, leading to the justification and reinforcement of one’s gambling behaviour (Ruiz de Lara et al., 2019). Nevertheless, it was posited that this ego-protective mechanism functions in tandem with gambling-related cognitive distortions and is most prominent amongst problem gamblers who are young and well-educated and have atypical reward sensitivity (Navas et al., 2019).

To illustrate, Ruiz de Lara et al. (2019) studied a sample of gamblers with different levels of gambling involvement and found that those with high cognitive reappraisal skills scored higher in gambling severity across all domains of cognitive bias except inability to stop. Moreover, gambling disorder patients with high cognitive bias were also found to have higher scores in positive refocusing—another customarily adaptive emotion strategy (Navas et al., 2016). As such, what remains to be investigated is the possible moderating role of gambling-related cognitive distortions in the relationship between cognitive reappraisal and problem gambling.

### Present Study

Although trait impulsivity and gambling-related cognitive distortions are well-established risk factors for problem gambling, discrepancies were noted regarding the role of cognitive reappraisal. Recent meta-analyses (Neophytou et al., 2023; Velotti et al., 2021) on cognitive reappraisal yielded mixed results that relate cognitive appraisal to both higher and lower levels of problem gambling, concluding with the suggestion that the nuances in this association might be better understood *vis-à-vis* other risk factors. Indeed, these inconsistencies in findings highlight the need to examine the possibility of moderator variables intervening in the observed relationship between cognitive reappraisal and problem gambling. Thus, the aims of this study are as follows:

1. To examine the associations between trait impulsivity, gambling-related cognitive distortions, cognitive reappraisal and problem gambling behaviour.
2. To examine the moderating effect of trait impulsivity on the association between cognitive reappraisal and problem gambling behaviour.
3. To examine the moderating effect of gambling-related cognitive distortions on the association between cognitive reappraisal and problem gambling behaviour.

## Method

### Participants

This study utilises a cross-sectional study design, and participants were recruited via convenience sampling. Electronic recruitment posters were disseminated on various social media platforms and online forums. Inclusion criteria for participation entail being a Malaysian who had engaged in gambling activity within the past year. Individuals who met the inclusion criteria were invited to complete the self-administered online Qualtrics survey. A total number of 157 responses were recorded. Six responses were removed due to missing data, i.e., less than 50% completion rate, whilst two multivariate outliers were excluded. The final number of participants is 149 adults (103 males, 46 females) aged between 20 and 59 years old (*M* = 32.18, *SD* = 9.58). Demographic information is presented in Table 1. The majority of participants are well-educated, working Chinese young adults who identify as male. As such, it must be noted that this is not a highly representative sample, and the subsequent results might only be applicable to individuals that fall within this cohort.

### Ethics

All procedures of the present study conformed to ethical standards for research involving human participants and APA ethical guidelines. The study methodology and design were approved by the Monash University Human Research Ethics Committee (Project ID: 28416). Participants were provided with an explanatory statement and were required to give their informed consent prior to participating in this study.

**Table 1:** Demographic information

Demographic categories	Frequency	Percentage (%)
**Sex**		
Male	103	69.12
Female	46	30.87
**Ethnicity**		
Chinese	107	71.81
Malay	4	2.68
Indian	8	5.37
Others	7	4.70
Undisclosed	23	15.44
**Highest Education Level**		
University or graduate degree	117	78.52
High school degree or other	32	21.48
**Employment Status**		
Student	27	18.12
Employed	105	70.47
Unemployed	11	7.38
Retired	6	4.03

### Measures

***South Oaks Gambling Screen*** (SOGS; Lesieur and Blume, 1993). Problem gambling behaviour was measured by the SOGS. The SOGS is a 20-item self-reported questionnaire that assesses gambling severity (e.g. “Did you ever gamble more than you intended to?”), gambling dependence (e.g. “When you gamble, how often do you go back another day to win back money you lost?”) and common gambling-related problems (e.g. “Have you ever lost time from work or school due to money or gambling?”). Items were scored either a one or a zero for each response, and the total score ranges from zero to twenty. Higher mean SOGS scores indicate higher problem gambling severity. Although the SOGS is known to overestimate the number of pathological gamblers amongst community and treatment-seeking gamblers (Tang et al., 2010), as well as the general population (Stinchfield, 2002), the purpose of utilising the SOGS in this study is to provide a continuous measure of problem gambling behaviour and not as a diagnostic or classification tool. In the current study, the SOGS demonstrated good reliability (Cronbach’s *α* = 0.85).

***Emotion Regulation Questionnaire-Cognitive Reappraisal Subscale*** (ERQ-CR; Gross and John, 2003). Cognitive reappraisal was measured by the ERQ-CR (e.g. “I control my emotions by changing the way I think about the situation I’m in”). The 6-item self-reported ERQ-CR is rated on a 7-point Likert scale ranging from 1 (strongly disagree) to 7 (strongly agree). Higher mean ERQ-CR scores indicate higher cognitive reappraisal skills. The ERQ has demonstrated good internal consistency and temporal stability (Gross & John, 2003; Sala et al., 2012), as well as sound convergent and discriminant validity (Gross & John, 2003). The ERQ is also often utilised to asses emotion regulation strategies in gambling research (Jara-Rizzo et al., 2019; Williams et al., 2012). In the current study, the ERQ-CR demonstrated good reliability (Cronbach’s *α* = 0.89).

***Short-UPPS-P Impulsive Behaviour Scale*** (SUPPS-P; Cyders et al., 2014). Trait impulsivity was measured by the SUPPS-P. The SUPPS-P is a 20-item self-reported questionnaire that examines the five facets of trait impulsivity, i.e., negative urgency (e.g. “When I am upset I often act without thinking”), lack of premeditation (e.g. “​​My thinking is usually careful and purposeful), lack of perseverance (e.g. “I finish what I start”), sensation seeking (e.g. “I quite enjoy taking risks”), and positive urgency (e.g. “I tend to lose control when I am in a great mood”). Items were rated on a 4-point Likert scale ranging from 1 (agree strongly) to 4 (disagree strongly). Items for negative urgency, positive urgency, and sensation seeking were reversed scored. Higher mean SUPPS-P scores indicate higher trait impulsivity. Psychometric studies on the SUPPS-P have confirmed that it replicates the original factor structures and the internal consistency of the original scale (Cyders et al., 2014, D’Orta et al., 2015). Validity of the SUPPS-P has been supported by expected relations found with depression and anxiety (D’Orta et al., 2015) and risky behaviours, such as problem gambling (Dugrè et al., 2019). In the current study, the SUPPS-P demonstrated excellent reliability (Cronbach’s *α* = 0.92).

***Gambling Related Cognitions Scale*** (GRCS; Raylu and Oei, 2004b). Gambling-related cognitive distortions were measured by the GRCS. The GRCS is a 23-items self-reported questionnaire that examines the five categories of gambling cognitions, i.e., gambling expectancies (e.g. “Gambling makes me happier”), illusion of control (“Praying helps me win”), predictive control (e.g. “When I have a win once, I will definitely win again”), inability to stop gambling (e.g. “I can’t function without gambling”), and interpretive bias (e.g. “Relating my winnings to my skill and ability makes me continue gambling”). Items were rated on a 7-point Likert scale ranging from 1 (strongly disagree) to 7 (strongly agree). Higher mean GRCS scores indicate higher gambling-related cognitive distortions. Psychometric studies also revealed that the GRCS measures have adequate internal consistency and possess good concurrent, predictive and criterion-related validity, and thus, it is a useful instrument for identifying GRC amongst non-clinical gamblers (Raylu & Oei, 2004a, b). In the current study, the GRCS demonstrated excellent reliability (Cronbach’s *α* = 0.94).

### Statistical Analyses

SPSS and R were used to conduct all statistical analyses. For data cleaning and assumption checks: outliers were identified, normality in the outcome variable was examined, variance inflation factor (VIF) for diagnosing multicollinearity was calculated, and model diagnostics for regression assumptions were conducted. For preliminary analyses, the mean, standard deviation and bivariate correlation coefficients for all the variables were calculated. For the main analyses, hierarchical linear regressions (HLR) and dominance analysis (Azen & Budescu, 2006) were conducted. Subsequently, simple slope analyses were used to probe the interaction effects. An alpha value of 0.05 was used as the cut-off for significance.

## Results

### Assumptions Checks

As multicollinearity is a concern in moderation regression, all the variables were mean-centred (Iacobucci et al., 2016). VIF for the HLR models fell within the acceptable range (≤ 3.00). Regression assumptions were examined using Model Diagnostics in R. Visual inspection of the Q-Q plots and scatter plots indicated that assumptions of normality in residuals and homogeneity of variance were not violated.

### Preliminary Analyses

The means, standard deviations, and correlation coefficients for all variables in this study are presented in Table 2. Sex and all three predictor variables were significantly correlated with the outcome variable. Age had a marginally significant correlation with the outcome variable. Being female and being older was negatively correlated with problem gambling, whilst impulsivity, gambling-related cognitive distortions and cognitive reappraisal skills were positively correlated with problem gambling.

**Table 2:** Descriptive statistics and zero-order correlations for all variables

	*Mean*	*SD*	Sex: F	Age	1	2	3	4
Sex: Female	-	-	1					
Age	32.18	9.58	0.09	1				
1. Impulsivity	2.40	0.55	− 0.36**	− 0.10	1			
2. Gambling cognitions	3.31	1.21	− 0.40**	− 0.05	0.81**	1		
3. Cognitive reappraisal	5.30	1.09	− 0.14**	− 0.06	0.29**	0.31**	1	
4. Problem gambling	0.24	0.19	− 0.42**	− 0.16*	0.70**	0.78**	0.27**	1

*Note.* Impulsivity was measured by SUPPS-P, gambling cognition was measured by GRCS, cognitive reappraisal was measured by ERQ-CR, and problem gambling behaviour was measured by SOGS. *p = .053, **p < .01

### Main Analyses

For Model 1, sex and age were entered in the first step of the HLR whilst impulsivity, gambling-related cognitive distortions and cognitive reappraisal were added in the second step. In the first stage, sex was a significant predictor of problem gambling. Of the three predictors entered in the second stage, only impulsivity (*β* = 0.18, *p* = .04), and gambling-related cognitive distortions (*β* = 0.58, *p* < .001) were significant predictors of problem gambling. The three predictors significantly improve the model prediction *ΔF* (3, 143) = 59.62, *p* < .001, and have a large effect size *ΔR*^*2*^ = 0.45. Dominance analysis with *R*^*2*^ as the fit index was also performed. The average contribution of gambling-related cognitive distortions was the highest at *R*^*2*^ = 0.32. Results from the HLR and the dominance analysis are presented in Table 3.

**Table 3:** Results of hierarchical linear regression for Model 1

	Step 1			Step 2			Dominance analysis
	*Std coef*	*t*	*95% CI*	*Std coef*	*t*	*95% CI*	*R* ^*2*^
Sex: Female	− 0.41***	-5.48	− 0.222, − 0.104	− 0.11*	-2.01	− 0.087, − 0.001	0.067
Age	− 0.12	-1.61	− 0.005, 0.001	− 0.10*	-2.03	− 0.004, 0.000	0.014
Impulsivity				0.18*	2.09	0.003, 0.120	0.216
Gambling cognition				0.58***	6.60	0.064, 0.118	0.321
Cognitive reappraisal				0.013	0.25	− 0.016, 0.021	0.023
*Adjusted R* ^*2*^	0.18			0.63			
*ΔR* ^*2*^	0.19			0.45			
*ΔF*	17.28***			59.62***			

*Note.* Predictor variables are impulsivity as measured by SUPPS-P, gambling cognition as measured by GRCS, and cognitive reappraisal as measured by ERQ-CR. Outcome variable is problem gambling behaviour as measured by SOGS. *p < .05, ***p < .001

For Model 2, sex and age were entered in the first step of the HLR, impulsivity and cognitive reappraisal were added in the second step, and the interaction term, impulsivity*cognitive reappraisal, was added in the third step. Results are presented in Table 4. The interaction term is a significant predictor of problem gambling (*β* = 0.17, *p* = .009) that significantly improves the model prediction *ΔF* (1, 143) = 7.04, *p* = .009, and has a small effect size *ΔR*^*2*^ = 0.02.

**Table 4:** Results of hierarchical linear regression for Model 2

	Step 1			Step 2			Step 3		
	*Std coef*	*t*	*95% CI*	*Std coef*	*t*	*95% CI*	*Std coef*	*t*	*95% CI*
Sex: Female	− 0.41***	-5.48	− 0.222 − 0.104	− 0.19**	-3.01	− 0.122, − 0.025	− 0.21***	-3.51	− 0.133, − 0.037
Age	− 0.12	-1.61	− 0.005, 0.001	− 0.07	-1.32	− 0.004, 0.001	− 0.05	-0.85	− 0.003, 0.001
Impulsivity				0.61***	9.52	0.166, 0.253	0.55***	8.36	0.145, 0.235
Cognitive reappraisal				0.07	1.11	− 0.009, 0.032	0.12*	1.92	− 0.001, 0.042
Impulsivity * Cognitive reappraisal							0.17**	2.65	0.013, 0.091
*Adjusted R* ^*2*^	0.18			0.52			0.54		
*ΔR* ^*2*^	0.19			0.34			0.02		
*ΔF*	17.28***			52.19***			7.04**		

*Note.* The predictor variables are impulsivity as measured by SUPPS-P, cognitive reappraisal as measured by ERQ-CR and the interaction term. The outcome variable is problem gambling behaviour as measured by SOGS. *p = .057, **p < .01, ***p < .001

**Fig. 1:**
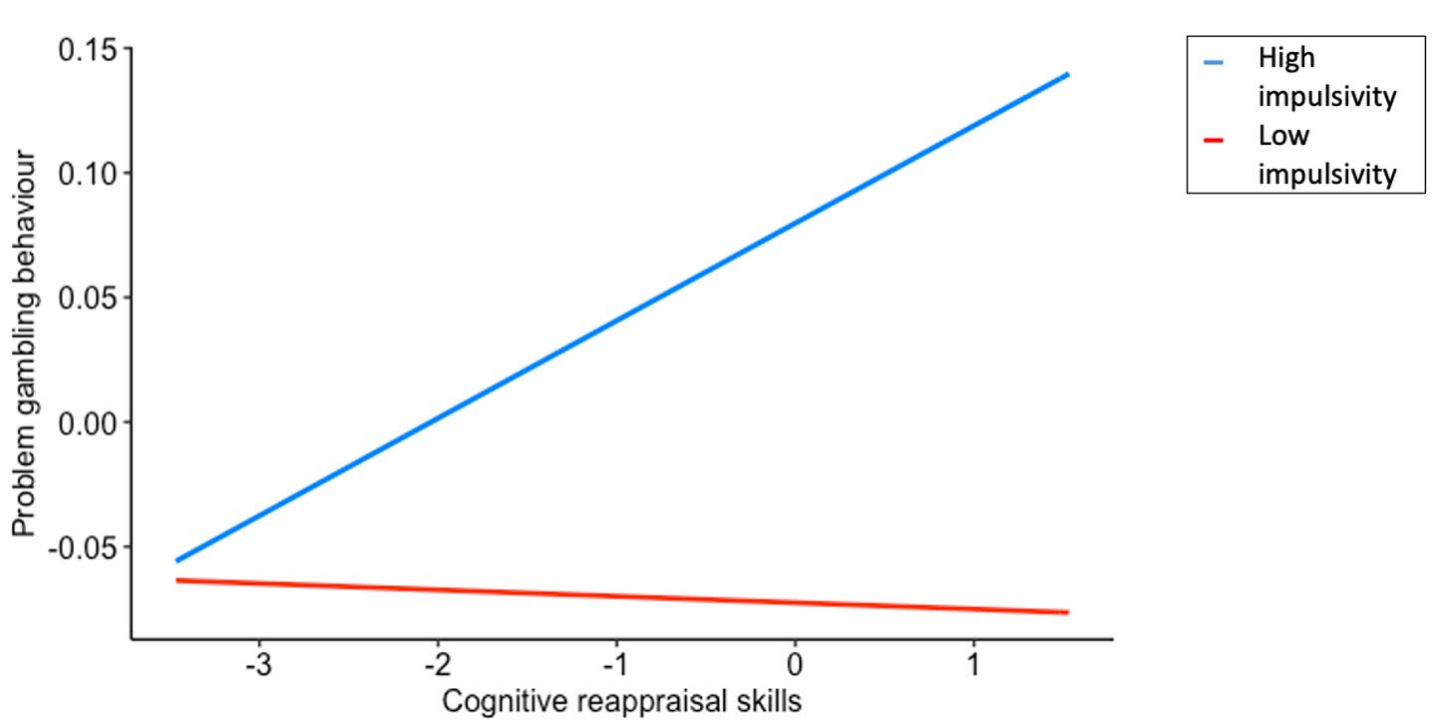
Problem gambling behaviour as a function of impulsivity and cognitive reappraisal skills *Note:* At high impulsivity, *b* = 0.19 [0.145, 0.235], *p* < .001. At low impulsivity, *b* = 0.19 [0.145, 0.235], *p* < .001

Simple slopes analysis indicated that the simple effects of cognitive reappraisal on problem gambling were significant at both high and low (+/- 1 *SD*) impulsivity. As depicted in Fig. 1, having high impulsivity strengthens the association between cognitive reappraisal and problem gambling in that at high impulsivity, there is a positive correlation between cognitive reappraisal and problem gambling but at low impulsivity, the correlation was approximately zero.

For Model 3, sex and age was entered in the first step of the HLR, gambling-related cognitive distortions and cognitive reappraisal were added in the second step, and the interaction term, gambling-related cognitions*cognitive reappraisal, was added in the third step. Results are presented in Table 5. The interaction term is a significant predictor of problem gambling (*β* = 0.16, *p* = .002) that significantly improves the model prediction *ΔF* (1, 143) = 10.11, *p* = .002, and has a small effect size *ΔR*^*2*^ = 0.02.

**Fig. 2:**
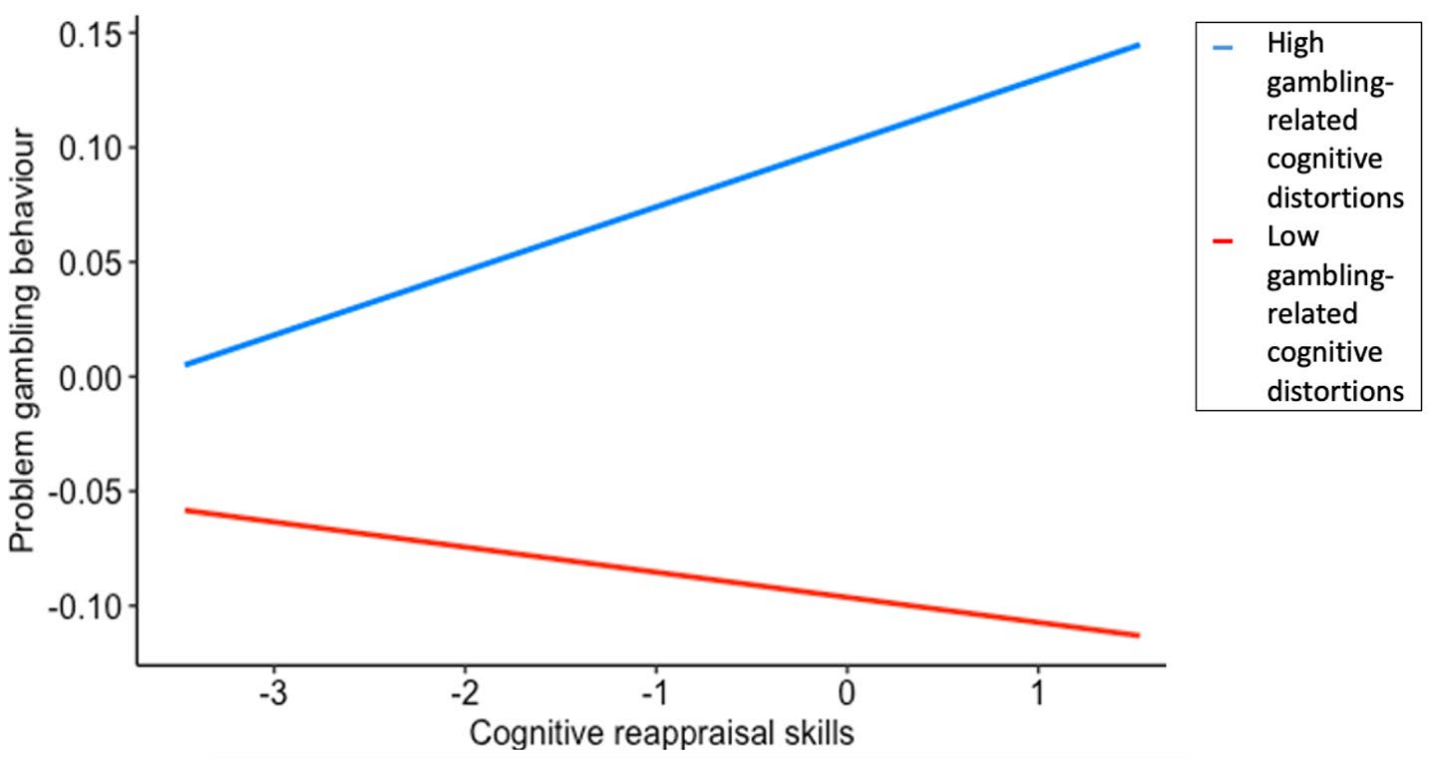
Problem gambling behaviour as a function of gambling-related cognitive distortions and cognitive reappraisal skills *Note:* At high gambling-related cognitive distortions, *b* = 0.11 [0.093, 0.128], *p* < .001. At low gambling-related cognitive distortions, *b* = 0.11 [0.093, 0.128], *p* < .001

Simple slopes analysis indicated that the simple effects of cognitive reappraisal on problem gambling were significant at both high and low (+/- 1 *SD*) gambling-related cognitive distortions. As depicted in Fig. 2, at high levels of gambling-related cognitive distortions, there is a positive correlation between cognitive reappraisal and problem gambling but at low levels of gambling-related cognitive distortions, cognitive reappraisal is negatively correlated to problem gambling.

**Table 5:** Results of hierarchical linear regression for Model 3

	Step 1			Step 2			Step 3		
	*Std coef*	*t*	*95% CI*	*Std coef*	*t*	*95% CI*	*Std coef*	*t*	*95% CI*
Sex: Female	− 0.41***	-5.48	− 0.222, − 0.104	− 0.12*	-2.11	− 0.090, − 0.003	− 0.12*	-2.29	− 0.092, − 0.007
Age	− 0.12	-1.61	− 0.005, 0.001	− 0.11*	-2.23	− 0.004, 0.000	− 0.12*	-2.36	− 0.004, 0.000
Gambling cognition				0.72***	12.38	0.095, 0.131	0.70***	12.49	0.093, 0.128
Cognitive reappraisal				0.02	0.36	− 0.015, 0.022	0.05	1.00	− 0.009, 0.028
Gambling cognition * Cognitive reappraisal							0.16**	3.18	0.008, 0.035
*Adjusted R* ^*2*^	0.18			0.62			0.64		
*ΔR* ^*2*^	0.19			0.44			0.02		
*ΔF*	17.28***			85.24***			10.11**		

*Note.* The predictor variables are gambling cognition as measured by GRCS, cognitive reappraisal as measured by ERQ-CR and the interaction term. The outcome variable is problem gambling behaviour as measured by SOGS. *p < .05, **p < .01, ***p < .001

## Discussion

This study examined the association between trait impulsivity, gambling-related cognitive distortions, cognitive reappraisal skills and problem gambling in a sample of Malaysian gamblers. The results revealed impulsivity and gambling-related cognitive distortions as significant predictors of problem gambling, with gambling-related cognitive distortions contributing the most to the prediction model. At high levels, impulsivity and cognitive distortions are also significant moderators that strengthen the association between cognitive reappraisal and problem gambling.

Impulsivity and gambling-related cognitive distortions recorded a significant positive association with problem gambling. Indeed, impulsivity manifests as the inability to regulate impulses and exercise delayed gratification, which is not only a hallmark of problem gambling symptoms (McCormick & Taber, 1987), but is also persistently observed in other forms of addiction, e.g., online gaming (Blinka et al., 2016), alcohol use (Shin et al., 2012), and cigarette smoking (Doran & Tulley, 2018). As for problem gambling, the role of impulsivity as a risk factor is evident across literature—ranging from longitudinal studies (Dussault et al., 2011; Liu et al., 2013) to meta-analyses (Browne et al., 2019; Johansson et al., 2009). Thus, findings from this study are consistent with gambling research.

Gambling-related cognitive distortions are also significantly associated with problem gambling. This finding is consistent with past research that identified gambling-related cognition as a risk factor for problem gambling (Mitrovic & Brown, 2009; Orgaz et al., 2013; Raylu & Oei, 2004b). Moreover, individuals with higher gambling-related cognitive distortions have been associated with higher participation in both skill-based and chance-based gambling (Mestre-Bach et al., 2021).

Gambling-related cognitive distortions had the largest effect size and contributed most to the variance in problem gambling behaviour amongst Malaysian gamblers. These findings could be attributed to cross-cultural differences. As the majority (71.81%) of participants in this study are Chinese, and gambling remains a popular pastime amongst the Chinese population around the world (Loo et al., 2008), it is possible that the majority of the participants in this study also hold higher positive expectations for gambling outcomes than the average gambling cohort. Moreover, as compared to Western populations, the Chinese population reported a higher prevalence of superstitious beliefs (Oei et al., 2008; Raylu & Oei, 2004a). It is possible that due to cultural values and norms, Chinese gamblers might engage more in gambling rituals to cultivate luck or prevent misfortunes. This practice could cause the illusions of control facet of gambling-related cognitive distortions to have a more salient effect on problem gambling.

Trait impulsivity is a significant moderator that strengthens the association between cognitive reappraisal and problem gambling. The effects are such that there is a positive correlation between cognitive reappraisal and problem gambling at high impulsivity, but at low impulsivity, the correlation was approximately zero. As Frijda et al. (2014) argued, impulsivity as a response to an unpleasant stimulus is associated with a certain degree of urgency that supersedes reasoning. Accordingly, individuals with high trait impulsivity have been shown to have difficulty accepting emotional states in a non-reactive and non-judgemental manner (Peters et al., 2011). This difficulty in detaching oneself from the situation and remaining objective in the face of adverse stimuli may alter cognitive reappraisal attempts (Webb et al., 2012). As such, high impulsivity might not only be associated with high gambling severity but also have a role in disrupting reappraisal skills.

When gambling becomes habituated as a maladaptive coping mechanism, problem gamblers are confronted with increasing amounts of gambling loss. Having high impulsivity and, thus, lower objectivity could then influence these individuals to alter their emotional reactions towards gambling outcomes. Through cognitive reappraisal, emotional information that would typically contribute toward refraining from future gambling activities (e.g. shame and guilt attached to events of gambling losses) are rationalised and reinterpreted (Yi & Kanetkar, 2011). The rationalisation process also interferes with the development of a negative heuristic of monetary loss due to gambling and, thus, contributes towards the exacerbation of gambling severity (Clark, 2010). This reasoning is also coherent with Gross’s (2014) model of emotion, which posits that emotions are only actively regulated and modified when they interfere with one’s desired behaviour or goals. Indeed, given that the hallmarks of problem gambling include the over-representation and over-valuation of gambling as an emotion regulation strategy (Rogier & Velotti, 2018); amongst gamblers with high impulsivity, utilising reappraisal skills to mitigate the effects of inevitable losses could take precedence over applying said skills, instead of gambling, to cope with aversive emotional states.

The moderating effects of gambling-related cognitive distortions are that cognitive reappraisal is positively associated with higher problem gambling at higher levels. At lower levels, cognitive reappraisal is negatively associated with problem gambling. From these findings, it could be understood that amongst gamblers with low gambling-related cognitions, cognitive reappraisal skills functions as it is traditionally understood to be—an adaptive form of emotion regulation strategy that down-regulates negative emotions—and is associated with lower problem gambling. Instead of engaging in maladaptive behaviours (e.g. gambling) to escape their negative emotions, individuals in this category exercise cognitive reappraisal to alter their emotional reactions (Teal et al., 2021). Thus, cognitive reappraisal skills might be a protective factor for problem gambling at low levels of gambling-related cognitions.

On the contrary, high gambling-related cognition distortions are tied to the biased reinterpretation of gambling-related events. This finding is congruent with past studies. For example, a study by Navas et al. (2016) found that problem gamblers with higher cognitive bias scored higher across a range of other emotion regulation strategies. These putatively adaptive strategies were also positively associated with problem gambling severity. Ruiz de Lara et al. (2019) found that high gambling-related cognitive distortions, in conjunction with reappraisal skills, can reframe gambling expectancies and outcomes in such a way that affords gamblers the continued justification of their gambling behaviour despite its negative consequences. Moreover, findings for this set of specific relationships between strong gambling-related cognitions, high cognitive reappraisal skills and increased problem gambling behaviour were also reflected in another paper by Jara-Rizzo et al. (2019).

### Limitations

The findings from this study must be considered in light of several limitations. Most glaringly, however, is the small sample size and the fact that the majority of participants are well-educated young Chinese men. Hence, it is likely that the findings may not be representative of problem gambling in other diverse populations, and the generalisability of the results must be confirmed by future studies with larger and more representative samples.

The cross-sectional design precludes the inference of causal relationships. And as participants consisted of community gamblers who reported lower problem gambling severity, generalising the findings of this study to a clinical cohort might not be plausible. This study also employed subjective self-report measures. Although the scales demonstrated good reliability in this study, gambling is considered a sensitive topic for some, and thus, responses might be influenced by social desirability bias. Lastly, for the sake of parsimony, this study did not consider the independent effects of each facet in the constructs of trait impulsivity and gambling-related cognitions.

### Future Directions

Future studies could employ an experimental design to ascertain cause-and-effect relationships. Alternatively, similar models with longitudinal study designs could also offer insight into the temporal relationship between variables. In addition, replication studies with larger sample sizes and a more diverse sample could afford more generalisability. Future research could also consider the use of behavioural measures of impulsivity (e.g. experimental tasks), which would allow researchers to determine if findings from this study extend to other domains of impulsivity. Lastly, it would be worthwhile to examine different gambling activities (i.e., skill-based gambling, chance-based gambling), specific gamblers’ profiles (i.e., emotionally vulnerable gamblers, self-deceptive gamblers, impulsive-antisocial gamblers), as well as the subscales of trait impulsivity, gambling-related cognitive distortions and emotion regulation. Doing so could provide more nuanced insight into these associations.

### Implications

Findings from this study endorse problem gambling interventions that tackle cognitive reappraisal in a contextualised, two-step manner. For impulsive gamblers and self-deceptive gamblers, impulsivity and gambling-related cognitions *vis-à-vis* the possible misapplication of cognitive reappraisal skills should be addressed. Moreover, the application of cognitive reappraisal skills is often spontaneous and occurs implicitly (Kobylińska & Kusev, 2019; Lambie & Marcel, 2002). And amongst problem gamblers, these automatic, over-practised emotion regulation strategies could potentially backfire by exacerbating problem gambling behaviour instead. As such, interventions to develop appropriate emotion regulation strategies amongst problem gamblers should adopt a context-specific approach.

Moreover, gambling-related cognitions might be especially pertinent to problem gamblers who are well-educated young Chinese men. Thus, interventions for this cohort should prioritise targeting distorted gambling cognitions. Indeed, developing intervention programs that have a more targeted approach is important because several papers (Aragay et al., 2015; Fortune & Goodie, 2012; Jara-Rizzo et al., 2019) have demonstrated that personal motivation, treatment compliance, retention rates, and intervention efficacy are generally less promising when the treatment does not address the relevant underlying factors.

## Conclusion

In conclusion, our results contribute to the understanding of cognitive reappraisal in gambling research and shed light on the roles of impulsivity and gambling-related cognitive distortions as moderators in the relationship between cognitive reappraisal and problem gambling. Specifically, our findings uncover the double-edged nature of cognitive reappraisal in problem gambling. At low levels of gambling-related cognitive distortions, reappraisal might be a protective factor. However, amongst gamblers with high impulsivity or high cognitive distortions, cognitive reappraisal could distort reality in a self-serving way and potentially exacerbate problem gambling severity by interfering with the emotional and motivational processes of problem gambling.

## Declarations

### Research Materials Statement

The data that support the findings of this study can be made available from the corresponding author upon request.
